# Perceived Parenting Style and Subjective Well-Being among Chinese Nursing Undergraduates: The Role of Self-Efficacy and Gender

**DOI:** 10.3390/ijerph191912654

**Published:** 2022-10-03

**Authors:** Haitao Huang, Haishan Tang, Guangli Lu, Chaoran Chen, Qianwen Peng, Yiming Zhang, Yipei Liang, Xiao Wan, Yueming Ding

**Affiliations:** 1 Institute of Nursing and Health, School of Nursing and Health, Henan University, Kaifeng 475004, China; 2 Institute of Business Administration, School of Business, Henan University, Kaifeng 475004, China

**Keywords:** parenting style, subjective well-being, self-efficacy, gender, undergraduate nursing students

## Abstract

The question of how to improve the subjective well-being (SWB) of nursing students is an important factor for reducing nursing loss and improving nursing quality. The current study aimed to investigate the influence of parenting style and self-efficacy (SE) on SWB among Chinese nursing undergraduates. The moderating role of gender between parenting style and SWB was also examined. Descriptive analysis, Pearson’s correlation analysis, and the Hayes’ PROCESS Macro Model 4 and Model 5 were used to analyze the available data. A total of 665 nursing undergraduates (M_age_ = 19.86, SD = 1.19) completed questionnaires. The results showed that PPS was positively correlated with SWB (r = 0.421, *p* < 0.01), while NPS was negatively correlated with SWB (r = −0.167, *p* < 0.01). Meanwhile, SE was positively correlated with PPS (r = 0.167, *p* < 0.01) and negatively correlated with NPS (r = −0.175, *p* < 0.01). In addition, SE was positively correlated with SWB (r = 0.273, *p* < 0.01) and played a partial mediating role in the association between parenting style and SWB. Furthermore, gender moderated the direct effect of parenting style on SWB. Specifically, compared with male nursing students, parenting style has a greater influence on the SWB of female nursing students. These findings can be used to develop targeted improvement strategies for nursing educators to improve SWB levels among nursing undergraduates.

## 1. Introduction

Due to the growing demand for nursing, unbalanced nurse-to-patient ratios, and increasing job pressures, it is becoming increasingly difficult to recruit and retain nurses globally, which is undermining nursing outcomes worldwide [1,2]. Therefore, there is an urgent need for us to train more nursing students. However, many nursing students do not consider nursing an interesting major for various reasons [3,4]. This leads to higher dropout rates and prevents us from producing more professional and enthusiastic nurses [5]. In this case, improving the subjective well-being (SWB) is considered to be one of the most effective ways to reduce student attrition [6]. SWB is an important indicator for measuring the positive degree of psychological development, involving an overall evaluation of the individual’s quality of life according to their own standards [7,8]. With the rise of positive psychology, an increasing number of studies have begun to concentrate on the positive effect of SWB and to explore the universal factors affecting SWB and the unique factors in different cultural and social situations [7]. Many studies have shown that SWB can not only improve academic performance and promote the mental health of nurses, but also improve creativity and organizational performance and reduce turnover rates [9,10,11]. Therefore, the maintenance of good mental health has become an important part of the training and development of future nurses.

As a backup force for clinical nurses, nursing students typically experience higher stress levels [12]. A survey from China conducted during the COVID-19 pandemic shows that nearly 20% of Chinese nursing students suffer from severe anxiety, and that more than 50% of nursing students are diagnosed with depression, which is higher than the rate for other Chinese teenagers [13]. It has been demonstrated that nursing students with higher levels of mental health are likely to be more productive in academic performance and clinical research. At the same time, they work more efficiently and provide better nursing to patients [14]. Therefore, improving the mental health of nursing students is essential for consolidating the nursing force. Although there have been previous international studies on the SWB of nursing students, few of these have involved Chinese nursing undergraduates [15,16]. The undergraduate stage is the key stage for the formation of the professional concept, value, and professional ability of nursing students [17]. Data from China show that nurses with advanced diplomas or bachelor’s degrees are the most needed members of the workforce at all levels of health care and in the primary care sector [18,19]. In view of the above considerations, it is essential to investigate SWB and its related factors among Chinese nursing undergraduates, which may offer significative guidance for future education.

### Background

The study of SWB has grown immensely over the past several decades, from it being a very small backwater area with few studies to a large endeavor occupying many scientists across different fields [7]. Diener believes that SWB is an individual’s overall judgment of their quality of life based on self-internalized standards and has the characteristics of subjectivity, integrity, and stability [20]. It includes both reflective cognitive judgements (such as life satisfaction) and emotional responses to ongoing life in terms of positive and pleasant emotions versus unpleasant and negative emotions [21]. When people reflect on their lives and make judgements about their life as a whole, or about domains in their life such as work and health, they make comparisons with the standards they have for the good life [21]. The satisfaction of goals theories assumes that the satisfaction of key needs, desires, and goals will give rise to high levels of SWB, and that dissatisfaction with them will give rise to low levels of SWB. At present, the factors closely related to SWB can be divided into two types: external factors and internal factors [22]. External factors mainly include life events (such as immigration), family factors (such as parenting style), and peer factors (such as peer attachment), while internal factors mainly include individual personality traits (such as self-esteem, optimism) and emotional states (such as depression, anxiety), etc. Among the external factors, parenting style has attracted much attention from researchers [23]. The Ecological Systems Theory (EST) of human development proposed by American psychologist Bronfen Brenner undoubtedly provides a very valuable theoretical basis and theoretical perspective for research in this field [24]. The EST emphasizes the interaction of various factors within a system. As a complex micro-system, the family plays an important role in the growth and development of children [25]. Since parents still tend to be the most important people closely in students’ lives, their parenting style has an important influence on students’ behaviors, attitudes, and emotions [26]. Parenting style is the synthesis of parenting attitude, concept, and behavior, and reflects the nature of the parent-child relationship [27]. Recently, studies have confirmed that the character of parents has a direct influence on the SWB of individuals [28,29]. Furthermore, other papers have shown that parenting styles are significantly associated with anxiety and depression in college students [30]. However, results on the extent to which parenting styles can affect the SWB of nursing undergraduates in mainland China are still lacking. Given that nursing undergraduates are an important part of the future nursing force in China, it is essential to investigate the association between parenting styles and SWB.

SE relates to the degree of confidence in one’s ability to complete tasks, which indicates an individual’s affirmation of his ability and confidence in facing new challenges and new environments [31]. Many studies have shown that SE can promote physical and mental health [32,33]. According to Social Cognitive Theory (SCT), SE is significantly affected by the external environment [34]. As an important external environment, parenting style has an important impact on the SE of students at different stages [35]. SE not only refers to the motivation of the independent self, but also forms the basis for the independent self to achieve other goals and meet needs. SE has a significant predictive effect on SWB [36]. Individuals with higher SE are generally considered to have stronger self-confidence, and this strong self-confidence will generate positive emotions, thereby enhancing SWB [36]. In addition, people with high SE usually attribute behavioral success to their own abilities and efforts, and attribute failure to insufficient effort or external uncontrollable factors [37]. This attribution method leads to improved motivation and reduced anxiety [37]. However, whether SE plays a mediating effect in the association between parenting style and SWB has not yet been investigated.

**Figure 1:**
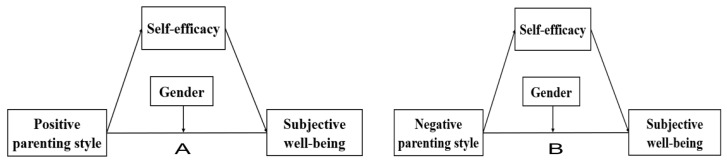
The proposed theoretical model.

According to the Gendered Family Process Model (GFPM), men and women are raised differently by their parents, and different parenting styles affect men and women differently [38]. At the same time, gender may also be an important demographic factor affecting SWB. Based on the results of 2907 participants, Cheng et al. indicated that the average score of SWB in female elderly was significantly higher than that in male elderly [39]. Other studies have indicated that women tend to have higher levels of emotional distress and are more susceptible to entering negative relationships than men [40]. In addition, studies have demonstrated that gender moderates the association between college students’ parenting styles and the Zhongyong Thinking Style [30]. However, to the best of our knowledge, no study has yet investigated whether gender moderates the association between parenting styles and nursing students’ SWB. Therefore, based on Ecological Systems Theory, Social Cognitive Theory and Gendered Family Process Model, this study tests the following hypotheses (Figure 1):

**H1.**  *Positive parenting style (PPS) should show a positive correlation with SE, while negative parenting style (NPS) should show a negative correlation with SE.*

**H2.**  *PPS should show a positive correlation with SWB, while NPS should show a negative correlation with SWB.*

**H3.**  *SE should positively correlate with SWB; the higher the SE is, the higher the SWB should be.*

**H4.**  *SE should play a partial mediating role in both the association between PPS and SWB and the association between NPS and SWB.*

**H5.**  *Gender should play a moderating role in both the association between PPS and SWB and the association between NPS and SWB.*

## 2. Materials and Methods

### 2.1. Participants and Procedure

A convenience sampling method was used to recruit nursing undergraduates from two undergraduate universities in Henan, PR China from March to May 2022. Participants meet the following inclusion criteria: (1) full-time nursing undergraduates in Grade 1, Grade 2 and Grade 3; (2) Know the purpose of the research and volunteer to participate in the research; Additionally, the exclusion criteria were students who did not complete all questionnaires for various reasons. All the participants who met the inclusion criteria were given questionnaires. Equation N = 4Uα^2^S2/δ^2^ [41] was used to calculate the sample size. S = 0.56 was calculated from the pre-survey, the allowable error δ was set to 0.1, and α was set to 0.05, so N = 4 × 1.96^2^ × 0.56^2^/0.1^2^ = 482. Taking into account the sampling error and the possibility of invalid questionnaires, we distributed a total of 700 questionnaires. Finally, after removing 35 unqualified questionnaires, a total of 665 valid questionnaires were obtained.

Before sampling, we discussed the contents and procedures of the questionnaire with the psychological services departments involved in each university. Investigators were to begin handing out paper questionnaires to students as they gathered in a classroom (about 50 students at a time). Participants were not given any incentive or inducement throughout the test. Furthermore, participants were told that their answers to the questionnaire would be anonymous and confidential, and that the data collected would only be used for academic study.

### 2.2. Ethical Considerations

This study was reviewed and approved by Institutional Review Board of Henan Provincial Key Laboratory of Psychology and Behavior (reference: 20220107001) and was conducted in accordance with the Declaration of Helsinki.

### 2.3. Variable Measurements

Contents of the questionnaire used in the manuscript are provided in Supplementary Materials.

#### 2.3.1. The General Information Questionnaire

A demographic information instrument assessed participants’ characteristics including age, gender, and home location, etc.

#### 2.3.2. Chinese Version of Parenting Bonding Instrument (PBI)

The PBI was compiled by Parker [42] and modified by Yang [43], and was used to measure individuals’ subjective feelings about their parenting style. The scale consisted of 46 items and included three dimensions, care, control, and encouraging autonomy, in which care and encouraging autonomy were classed as positive parenting style, and control was classed as negative parenting style. The scale adopted the Likert 4-point scoring (1 to 4, respectively, indicate very inconsistent to very consistent). The revised Chinese version of PBI has been shown to have a good internal consistency in Chinese college students [43]. In our study, the Cronbach’s coefficients for positive parenting style dimension and negative parenting style dimension were 0.920 and 0.812, respectively.

#### 2.3.3. General Self-Efficacy Scale (GSEC)

The GSEC was chosen to measure self-efficacy [44]. The scale is a one-dimensional scale with 10 items, such as “If I try my best, I can always solve the problem”. A Likert 4 points scale was used to score from 1 to 4 points, which indicate from “completely inconsistent” to “completely consistent”, respectively. We calculated the average scores for all items, and the higher the score, the higher the SE was. The Chinese version of GSEC has a favorable internal consistency [45]. The internal consistency in our study was 0.882.

#### 2.3.4. Index of Well-Being (IWB)

The IWB was compiled by Campbell et al. [46]. IWB mainly measures the degree of happiness that participants are currently experiencing. The scale consists of two parts: the index of general affect and the index of life satisfaction. The former consists of 8 items and the latter has 1 item. All items have a 7-point rating and the weight of index of life satisfaction is 1.1. The SWB score is obtained by adding the index of general affect and the index of life satisfaction score. The higher the score is, the higher the SWB is. So far, research has demonstrated that the scale has good reliability and validity in Chinese college students [47]. In our study, the internal consistency of the instrument was 0.939.

### 2.4. Data Analysis

All the data were analyzed using IBM SPSS statistics 25.0 and the PROCESS macro3.3. PROCESS provides ordinary least-squares, regression-based path analysis such as structural equation modeling but supplies additional useful statistics and safeguards against irregular sampling distributions [48]. The demographic characteristics of the participants were represented by descriptive statistics. Pearson correlation analysis was used to investigate the association between PPS, NPS, SE and SWB. Harman’s single-factor test was used to examine the common method bias derived from self-reported data [49]. The mediating role of SE was examined using PROCESS Model 4. The moderating effect of gender was analyzed using PROCESS Model 5 [48]. In addition, we used the 5000 resample bootstrapping method with a 95% CI to test the effect of the independent variables on the dependent variable through the mediating variable. All *p* values were two-sided, with *p* < 0.05 indicating a statistically significant result. The report of this study is strictly in accordance with the STROBE Statement [50].

### 2.5. Validity and Reliability/Rigour

Firstly, all the instruments used in this study were adjusted in line with Chinese culture and verified to a have good validity and reliability (PBI-Positive: 0.920; PBI-Negative: 0.812; GSEC:0.882; IWB: 0.939). In addition, before the formal investigation, all investigators were trained on registration, checking the completeness of questionnaires, and the ethical tenets of conducting research. To reduce the risk of self-reported bias, the identities of all participants were kept strictly confidential. Finally, to ensure the rigor and accuracy of the statistical analysis, we invited a statistics professor to examine the data processing.

## 3. Results

### 3.1. Common Method Biases Tests

Harman’s single-factor test extracted 14 factors with eigenvalues greater than 1. The first factor explained 16.732% of the total variance, which is below the recommended threshold of 40% [49]. This suggests that common method bias is unlikely to confuse the data analysis results.

### 3.2. Participants’ Characteristics

A total of 665 undergraduate nursing students participated in and effectively filled out the questionnaire, including 149 males (22.4%) and 516 females (77.6%). The basic characteristics of the participants are shown in Table 1.

### 3.3. Descriptive Analysis and Correlations between Overall Variables

Table 2 shows the means, standard deviations (SD) and Pearson correlations of each variable. The average score for PPS was (2.564 ± 0.451), for NPS was (1.698 ± 0.293), for SE was (2.504 ± 0.519), and for SWB was (5.863 ± 0.627). As can be seen from the range of item scores, except for NPS, the scores of the remaining variables were basically at the medium level.

Pearson correlation analysis demonstrated that PPS was positively correlated with SWB (r = 0.421, *p* < 0.01), while NPS was negatively correlated with SWB (r = −0.167, *p* < 0.01). SE was positively correlated with PPS (r = 0.167, *p* < 0.01) and negatively correlated with NPS (r = −0.175, *p* < 0.01). In addition, SE was positively correlated with SWB (r = 0.273, *p* < 0.01). 

### 3.4. Testing the Mediation Effect of Self-Efficacy

Firstly, multiple linear regression analysis showed that gender and family structure had a significant influence on SWB. As a result, they were included as covariates in the mediation analysis.

Secondly, PROCESS Macro model 4 was used to analyze the mediating role of SE. PPS can significantly positively predict SWB after controlling for gender and family structure (c = 0.421, t = 11.924, *p* < 0.001). When PPS and SE were used in the regression equation together, the predictive effect of PPS on SWB was still significant (c′ = 0.383, t = 10.995, *p* < 0.001). PPS had a significant positive predictive effect on SE (a = 0.169, t = 4.524, *p* < 0.001), and SE had a significant positive predictive effect on SWB (b = 0.222, t = 6.226, *p* < 0.001). This manifested in SE partially mediating the relationship between PPS and SWB. The Bootstrap method test with percentile bias correction indicated that SE had a significant mediating effect between PPS and SWB, with ab = 0.038, Boot SE = 0.012, and 95%CI= (0.016, 0.064). 

**Figure 2:**
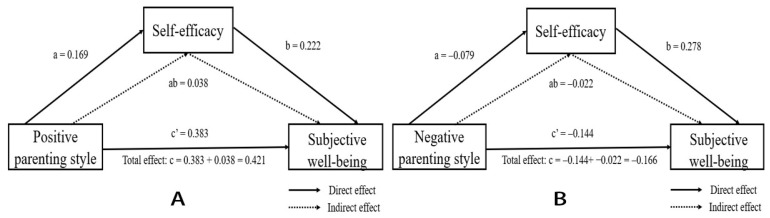
The mediating effect of SE ((**A**): the mediating effect of SE between PPS and SWB; (**B**): the mediating effect of SE between NPS and SWB).

NPS significantly negatively predicted SWB after controlling for gender and family structure (c = −0.166, t = −4.335, *p* < 0.001). When NPS and SE entered the regression equation together, the negative effect of NPS on SWB was still significant (c′ = −0.144, t = −3.893, *p* < 0.001). NPS had a significant negative predictive effect on SE (a = −0.079, t = −2.098, *p* < 0.001), and SE had a significant positive predictive effect on SWB (b = 0.278, t = 7.335, *p* < 0.001). This manifested in that SE partially mediated the relationship between NPS and SWB. The Bootstrap method test with percentile bias correction indicated that SE had a significant mediating effect between NPS and SWB, with ab = −0.022, Boot SE = 0.012, and 95%CI = (−0.046, −0.005). The contribution rates of indirect effects in the total effect were ab/(ab + c′)= (−0.022/−0.166)= 13.25%. Therefore, Hypotheses 1–4 were supported. Figure 2 shows the direct, indirect, and total effects. 

### 3.5. The Moderating Effect Analysis

**Figure 3:**
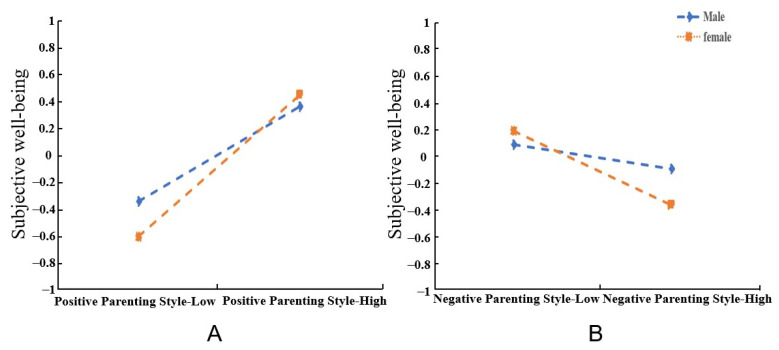
The moderating role of gender. ((**A**): the moderating role of gender between PPS and SWB; (**B**): the moderating role of gender between NPS and SWB).

PROCESS Macro model 5 was used to analyze the moderating role of gender. As shown in Table 3 and Table 4, the interaction terms between PPS and Gender (*β* = 0.169, *p* < 0.05) and the interaction terms between NPS and Gender (*β* = −0.195, *p* < 0.05) had significant predictive influence on the SWB of undergraduate nursing students after controlling for family structure. This suggests that gender moderates the association between parenting style and SWB. Therefore, Hypotheses 5 is supported. Simple slope analysis was used to further visually investigate the moderating role of gender. The results showed that the predictive effect of PPS on female SWB was significantly higher than that of PPS on male SWB (Female: *β* = 0.526, *p* < 0.001; Male: *β* = 0.354, *p* < 0.001). At the same time, a negative relationship between NPS and SWB was statistically significant among females (*β* = −0.280, *p* < 0.001) and males (*β* = −0.092, *p* < 0.05), but the effect size was much smaller among males. Figure 3 has shown the moderating role of gender between parenting style and SWB.

## 4. Discussion

Our study aimed to investigate the relationship between parenting style, SE, and SWB and determine the moderating effect of gender on the relationship between parenting style and SWB among nursing undergraduates in China. First, the results showed that there was a significant correlation between each of the two variables. Second, PPS and NPS were found to have a significant effect on SWB, and SE played a partial mediating role in the relationship. In the end, our study confirms that gender has a moderating effect in the association between parenting style and SWB. Specifically, compared with male nursing students, parenting style has a greater influence on the SWB of female nursing students. These attempts have important significance and practical value for the further research and improvement of the SWB of undergraduate nursing students. 

In this study, the PPS score obtained was higher than that in Deng‘s study, which was conducted in 2006, and the NPS is lower than that in Deng’s [51]. A possible explanation for this is that, on the one hand, the continuous implementation of the “A five-year plan on the guidance and development of family education” issued by the Chinese government has produced positive effects [52]. In addition, with the development of society and economy, Chinese families now pay more and more attention to scientific family education, which is manifested by parents’ increasing with concern, understanding of and respect for their children [52]. On the other hand, due to the emergence of new scientific evidence on child development and cultural shifts in the parent-child relationship, the norms of parenthood have changed considerably, with increasing emphasis placed on active parental involvement and effective interaction in children’s lives [53]. This may be an important reason for the improvement of PPS. The SE is at a medium level, which is consistent with the previous results [54], indicating that the participants had certain positive psychological resources, but that these still need to be strengthened. The SWB obtained was higher than Zou’s result [55], which may be related to the fact that the research object of the latter was registered nurses in the clinical work environment, while the investigation object of this study was the nursing students who have not yet entered the clinical work environment. Nursing students at school have not yet felt the pressure of the clinical work environment, so their SWB may be relatively high.

Our study confirmed that PPS was positively correlated with SWB, NPS was negatively correlated with SWB, and SE was significantly positively correlated with SWB. This is consistent with the results of previous studies [56,57] which enriches the literature on the antecedents of undergraduate nursing students’ SWB. According to the research of Xie [52], as one of the important contents of family system theory, parenting style was found to be related to many outcomes, such as academic performance and mental disorders, etc. At present, many scholars have recognized the importance of external environmental factors to SWB. They found that marital quality, economic status [58] and sociopolitical factors [59] were significantly associated with SWB. In addition, Croy et al. argue that SE is critical to the success of nursing students [60]. Students with high SE may have higher self-confidence when faced with academic tasks and are more likely to have a higher sense of accomplishment and SWB in their daily life [60]. However, the relationship between the parenting style, SE and SWB of undergraduate nursing students is rarely discussed. Our study shows that PPS was positively correlated with SWB, NPS was negatively correlated with SWB, and SE was significantly positively correlated with SWB, which adds to the literature on the antecedents of SWB among nursing undergraduates. 

Our findings demonstrated that PPS was positively correlated with SE and NPS was negatively correlated with SE; this is consistent with the results of a previous study [61]. This can be explained by the fact that PPS is beneficial to the formation of SE in nursing students. As an important part of the self-perception, SE is deeply influenced by the parenting style [31]. Nursing students with higher PPS were more likely to feel support from their families. Nursing students with a higher NPS are more likely to lack self-confidence and deny and belittle themselves excessively, which in turn leads to lower SE. 

This study proved that the parenting style of nursing students not only directly affected SWB, but also indirectly affected SWB through the partial mediating role of SE, which opened the “black box” of association between parenting style and undergraduate nursing students’ SWB. SE is considered a protective factor for individuals and is gradually cultivated in personal experience, including parenting styles [31]. Individuals view themselves according to their parents’ attitudes and evaluations, thereby forming their SE. SE also has a stable impact on social attitudes, making people look at things with a positive attitude and thereby improving SWB [36]. Bandura believes that the level of SE determines the emotional state and efficiency of activities [62]. However, no previous study has explored the mediating effects of SE between parenting style and SWB among undergraduate nursing students. Our study addresses this question and finds that parenting style is not only directly related to SWB in nursing undergraduates but also indirectly related to SWB through a partial mediating effect of SE. Taken together, this study found a new mediating mechanism to explain the association between parenting styles and SWB. 

Finally, this study found that gender moderated the direct association between parenting style and undergraduate nursing students’ SWB. Specifically, compared with male nursing students, parenting style had a greater impact on the SWB of female nursing students. Gender Schema Theory [63] and Gendered Family Process Model [38] show that there are significant gender differences in the impact of family on individual psychosocial development. The expectations of parents and society will cause parents to adopt different parenting styles for different genders. In the context of traditional Chinese culture, boys are encouraged by their parents to be independent, capable, and assertive, while girls are taught to be kind, gentle, and obedient [64]. Parents’ attitudes and gender role expectations will subtly affect the individual’s gender-role concept, thereby affecting coping style and personality [64]. Prior studies have shown that women are more prone to emotional arousal and empathy than men [65]. In addition, the process of women’s socialization is more reflected in the family emotional connection in China, and they are more inclined to maintain a closer relationship with their family [64]. Therefore, the SWB of Chinese female nursing students may be more influenced by parenting style. In conclusion, this study reveals a moderating effect of gender, which will contribute to the understanding of gender differences in the influence of parenting styles on SWB.

## 5. Limitations

Although there are good points to this study, several limitations must be considered. First of all, this study was a cross-sectional study, so further longitudinal studies are needed to investigate the causal association. Secondly, the data used in current research were all self-reported by the participants, which may have affected the results through recall bias. Although the deviation from common methods was not found in this study, we can still use a variety of data collection methods (such as the combination of self-report and report by others) in future studies to ensure the reliability of our conclusions. Finally, the participants of this study are only from two undergraduate universities, which hinders the promotion of the conclusions to some extent. Future studies can expand their sample sources and explore the differences in results obtained in different cultural backgrounds and educational levels.

## 6. Conclusions

In the present study, we aimed to investigate the relationship between parenting style, SE and SWB among Chinese nursing undergraduates. The moderating role of gender between parenting style and SWB was also examined. We found that parenting style and SE are important factors affecting the SWB of undergraduate nursing students, and that SE plays a partial mediating role between parenting style and SWB. In addition, gender played a moderating effect in the direct effect of parenting style on SWB. In view of these findings, this study possesses certain theoretical significance and practical value for improving the SWB of undergraduate nursing students. To improve SWB, the following suggestions are made. First, in order to increase the confidence of undergraduate nursing students in working in nursing after graduation, we recommend that nursing educators and the families of nursing students work together to improve the SWB of nursing students. The parents of nursing students should adopt a positive and democratic parenting style. For nursing educators, targeted interventions should be made for students with higher NPS levels, such as increasing students’ social support, talking with students frequently, etc. to enhance their immunity against psychological problems and improve their mental health, and then enhance their SWB.

Second, nursing educators can take various measures to improve the SE of nursing students, thereby promoting the improvement of SWB. For example, studies have shown that the more information students have about their major, the stronger their SE will be [66]. Therefore, nursing educators should focus on the improvement of their professional knowledge and skills and attract nursing students to the learning process through their rich knowledge reserves and excellent teaching skills. In addition, nursing educators can also improve the SE of nursing students through group interviews and interest courses. Finally, the findings indicated that gender moderates the direct effect of parenting styles on SWB. Therefore, we suggest that nursing educators need to comprehensively consider the differences in the psychological development characteristics of boys and girls when formulating strategies to improve the SWB of nursing students.

## Figures and Tables

**Table 1:** Demographic characteristics of undergraduate nursing students.

Characteristic		*N* = 665	%
Gender, n (%)			
	Male	149	22.4
	Female	516	77.6
Age, M (SD)		19.86 (1.19)	
Grade			
	Grade 1	275	41.4
	Grade 2	233	35.0
	Grade 3	157	23.6
Home Location			
	Town	244	36.7
	Village	421	63.3
Only child in family			
	Yes	93	14
	No	572	86
Monthly household income			
	<3000 RMB	242	36.4%
	3000–6000 RMB	338	50.8%
	>6000 RMB	85	12.8%

**Table 2:** Means, standard deviations and Pearson correlations of each variable.

Variables	Range	Mean±SD	1	2	3	4	5	6
1. PPS	1–5	2.564 ± 0.451	1					
2. PSC	1–5	2.452 ± 0.511	0.943 **	1				
3. PSEA	1–5	2.686 ± 0.501	0.783 **	0.531 **	1			
4. NPS	1–5	1.698 ± 0.293	−0.310 **	−0.224 **	−0.370 **	1		
5. SE	1–4	2.504 ± 0.519	0.167 **	0.148 **	0.148 **	−0.175 *	1	
6. SWB	1–7	5.863 ± 0.627	0.421 **	0.418 **	0.291 **	−0.167 **	0.273 **	1

**Abbreviations:** PPS, Positive Parenting Style; PSC, Parenting Style-Care; PSEA, Parenting Style- Encouraging Autonomy; NPS, Negative Parenting Style; SE, Self-Efficacy; SWB, Subjective Well-Being. * *p* < 0.05; ** *p* < 0.01.

**Table 3:** The moderating role of gender in the relationship between PPS and SWB.

Predictive Variable	Model 1 (Criterion: SWB)		Model 2 (Criterion: SE)		Model 3 (Criterion: SWB)	
	*β*	*t*	*β*	*t*	*β*	*t*
Family structure	0.175	4.579 **	0.164	3.088 **	0.281	3.296 **
PPS	0.421	11.924 **	0.173	4.580 **	0.388	11.121 **
SE					0.217	6.059 **
Gender					0.270	4.091 **
PPS × gender					0.169	1.983 *
R^2^	0.177		0.060		0.228	
F	47.529 **		21.288 **		38.854 **	

**Abbreviations:** PPS, Positive Parenting Style; SE, Self-Efficacy; SWB, Subjective Well-Being; NPS, Negative Parenting Style. * *p* < 0.05; ** *p* < 0.001.

**Table 4:** The moderating role of gender in the relationship between NPS and SWB.

Predictive Variable	Model 1 (Criterion: SWB)		Model 2 (Criterion: SE)		Model 3 (Criterion: SWB)	
	*β*	*t*	*β*	*t*	*β*	*t*
Family structure	0.081	2.093 **	0.434	3.834 **	0.124	2.178 *
NPS	−0.166	11.924 **	−0.080	−2.099 *	−0.134	−3.620 **
SE					0.282	7.442 **
Gender					0.240	3.503 **
NPS × gender					−0.195	−2.356 *
R^2^	0.028		0.037		0.109	
F	6.380 **		12.740 **		16.107 **	

**Abbreviations:** PPS, Positive Parenting Style; SE, Self-Efficacy; SWB, Subjective Well-Being; NPS, Negative Parenting Style. * *p* < 0.05; ** *p* < 0.001.

## Data Availability

The data presented in this study are available on request from the corresponding author.

## Supplementary Materials

The following supporting information can be downloaded at: https://www.mdpi.com/article/10.3390/ijerph191912654/s1. S1: Questionnaire content.
