# Supplementary material for: Perceived Parenting Style and Subjective Well-Being among Chinese Nursing Undergraduates: The Role of Self-Efficacy and Gender
Source: Int J Environ Res Public Health. 2022 Oct 3;19(19):12654. doi: 10.3390/ijerph191912654 (PMC9566302; doi:10.3390/ijerph191912654)
Supplement: Supplementary file 1 [file ijerph-19-12654-s001.zip › ijerph-1895892-supplementary.pdf]

Questionnaire content (Chinese version)

亲爱的同学：

您好!非常感谢您的参与!本调查仅供护理学研究之用。问卷共有五个部分,请您认真阅读每道题并按照自己的真实想法答题,答案没有对错、好坏之分。谢谢!请根据你的实际情况，在符合您的答案上画“√”,请务必回答每一个问题，不要漏项。感谢你的配合,祝你学业顺利,心想事成!

1、您的性别：A 男性　　B 女性

2、您的年龄：________

3、您所在的年级　　A 大一　　B 大二　　C 大三　　D 大四

4、您的家庭所在地　　A 城市　　B 农村

5、您是否为独生子女　　A 是　　B 否

6、家庭经济情况（人均月收入）A ＜3000 元　B 3000~6000 元　C ≥6000 元

父母教养方式量表

指导语：此问卷用于填写人对父母的养育方式分别进行评价。请认真阅读下面的每个句子，根据您目前的状况，每道题选择一个最接近的数值打“√“。0 代表非常不符合，1 比较不符合，2 比较符合，3 非常符合

1、用温和友好的语气与我说话

	非常不符合	比较不符合	比较符合	非常符合
父亲	0	1	2	3
母亲	0	1	2	3

2、没有给我足够的帮助

	非常不符合	比较不符合	比较符合	非常符合
父亲	0	1	2	3
母亲	0	1	2	3

3、允许我做自己喜欢的事情

	非常不符合	比较不符合	比较符合	非常符合
父亲	0	1	2	3
母亲	0	1	2	3

4、情感上显得对我冷淡

	非常不符合	比较不符合	比较符合	非常符合
父亲	0	1	2	3
母亲	0	1	2	3

5、了解我的问题与担忧

	非常不符合	比较不符合	比较符合	非常符合
父亲	0	1	2	3
母亲	0	1	2	3

6、对我很疼爱

	非常不符合	比较不符合	比较符合	非常符合
父亲	0	1	2	3
母亲	0	1	2	3

7、喜欢让我自己拿主意

	非常不符合	比较不符合	比较符合	非常符合
父亲	0	1	2	3
母亲	0	1	2	3

8、不想我长大

	非常不符合	比较不符合	比较符合	非常符合
父亲	0	1	2	3
母亲	0	1	2	3

9、试图控制我做的每一件事

	非常不符合	比较不符合	比较符合	非常符合
父亲	0	1	2	3
母亲	0	1	2	3

10、侵犯我的隐私

	非常不符合	比较不符合	比较符合	非常符合
父亲	0	1	2	3
母亲	0	1	2	3

11、经常对我微笑

	非常不符合	比较不符合	比较符合	非常符合
父亲	0	1	2	3
母亲	0	1	2	3

12、似乎不明白我需要什么或想要什么

	非常不符合	比较不符合	比较符合	非常符合
父亲	0	1	2	3
母亲	0	1	2	3

13、让我自己决定自己的事情

	非常不符合	比较不符合	比较符合	非常符合
父亲	0	1	2	3
母亲	0	1	2	3

14、让我觉得自己是可有可无的

	非常不符合	比较不符合	比较符合	非常符合
父亲	0	1	2	3
母亲	0	1	2	3

15、在我心烦意乱的时候可以让我心情好起来

	非常不符合	比较不符合	比较符合	非常符合
父亲	0	1	2	3
母亲	0	1	2	3

16、不经常与我交谈

	非常不符合	比较不符合	比较符合	非常符合
父亲	0	1	2	3
母亲	0	1	2	3

17、试图让我觉得我离不开他（她）

	非常不符合	比较不符合	比较符合	非常符合
父亲	0	1	2	3
母亲	0	1	2	3

18、觉得没有他（她）在身边我就不能照顾好自己

	非常不符合	比较不符合	比较符合	非常符合
父亲	0	1	2	3
母亲	0	1	2	3

19、给我足够自由

	非常不符合	比较不符合	比较符合	非常符合
父亲	0	1	2	3
母亲	0	1	2	3

20、允许我自由外出

	非常不符合	比较不符合	比较符合	非常符合
父亲	0	1	2	3
母亲	0	1	2	3

21、对我保护过度

	非常不符合	比较不符合	比较符合	非常符合
父亲	0	1	2	3
母亲	0	1	2	3

22、从不夸奖我

	非常不符合	比较不符合	比较符合	非常符合
父亲	0	1	2	3
母亲	0	1	2	3

23、允许我随心所欲地选择穿着

	非常不符合	比较不符合	比较符合	非常符合
父亲	0	1	2	3
母亲	0	1	2	3

主观幸福感量表

指导语：请认真阅读下面的每个条目，每个条目两端分别有代表两个极端的短语，数字 1-7 分别代表两个极端之间的 7 个等级，请根据您目前的状况，在每个条目上选择一个适合您的等级数值并打“√“。

1	厌倦的	1	2	3	4	5	6	7	有趣的
2	痛苦的	1	2	3	4	5	6	7	快乐的
3	无用的	1	2	3	4	5	6	7	有价值的
4	孤独的	1	2	3	4	5	6	7	朋友很多的
5	空虚的	1	2	3	4	5	6	7	充实的
6	无望的	1	2	3	4	5	6	7	充满希望的
7	沮丧的	1	2	3	4	5	6	7	有奖励的
8	生活未给予我任何机会	1	2	3	4	5	6	7	生活对我太好了
9	我对生活总体十分不满	1	2	3	4	5	6	7	我对生活总体十分满意

自我效能感量表

指导语：请认真阅读下面的每个句子，根据您目前的状况，每道题选择一个最接近的数值打“√“。1 代表完全不正确，2 有点正确，3 多数正确，4 完全正确

序号	题目	完全不正确	有点正确	多数正确	完全正确
1	如果我尽力去做的话，我总能解决问题的。	1	2	3	4
2	即使别人反对我，我仍然有办法获得我所要的。	1	2	3	4
3	对我来说，坚持理想和达到目标是轻而易举的。	1	2	3	4
4	我自信能有效地应付任何突如其来的事情。	1	2	3	4
5	以我的才智，我定能应付意料之外的情况。	1	2	3	4
6	如果我付出必要的努力，我一定能解决大多数难题。	1	2	3	4
7	我能冷静地面对困难，因为我相信自己处理问题的能力。	1	2	3	4
8	面对一个难题时，我通常能找到几个解决办法。	1	2	3	4
9	有麻烦的时候，我通常能想到一些应付的方法。	1	2	3	4
10	无论什么事在我身上发生，我都能应付自如。	1	2	3	4

Questionnaire content (English version)

Dear classmates:

Hello! Thank you very much for your participation! This survey is for nursing research purposes only. Please read each question carefully and answer according to your own real thoughts. There is no right or wrong, good or bad answer. Thank you! Please mark "√" on the answer that matches yours according to your actual situation. Please be sure to answer each question without missing items. Thank you for your cooperation, I wish you success in your studies and all your wishes come true!

1、Your gender: A male B female

2、your age: ______________

3、your grade A grade 1 B grade 2 C grade 3 D grade 4

4、your home location A town B rural

5、Are you an only child A yes B no

6、Family economic situation (per capita monthly income)

A ＜3000RMB B 3000~6000 RMB C ≥6000 RMB

Chinese version of Parenting Bonding Instrument

Guidance: This questionnaire is used to evaluate the parent's parenting style. Please read each sentence below carefully. According to your current situation, choose the closest value for each question and tick "√". 0 is very inconsistent, 1 is relatively inconsistent, 2 is relatively consistent, and 3 is very consistent

1、Spoke to me in a warm and friendly

	very inconsistent	relatively inconsistent	relatively consistent	very consistent
Father	0	1	2	3
Mother	0	1	2	3

2、Did not help me as much as need

	very inconsistent	relatively inconsistent	relatively consistent	very consistent
Father	0	1	2	3
Mother	0	1	2	3

3、Let me do those things i liked doing

	very inconsistent	relatively inconsistent	relatively consistent	very consistent
Father	0	1	2	3
Mother	0	1	2	3

4、Seemed emotionally cold to me

	very inconsistent	relatively inconsistent	relatively consistent	very consistent
Father	0	1	2	3
Mother	0	1	2	3

5、Appeared to understand my problems and worries

	very inconsistent	relatively inconsistent	relatively consistent	very consistent
Father	0	1	2	3
Mother	0	1	2	3

6、Was affectionate to me

	very inconsistent	relatively inconsistent	relatively consistent	very consistent
Father	0	1	2	3
Mother	0	1	2	3

7、Liked me to make my own decisions

	very inconsistent	relatively inconsistent	relatively consistent	very consistent
Father	0	1	2	3
Mother	0	1	2	3

8、Did not want me to grow up

	very inconsistent	relatively inconsistent	relatively consistent	very consistent
Father	0	1	2	3
Mother	0	1	2	3

9、Tried to control everything I did

	very inconsistent	relatively inconsistent	relatively consistent	very consistent
Father	0	1	2	3
Mother	0	1	2	3

10、Invaded my privacy

	very inconsistent	relatively inconsistent	relatively consistent	very consistent
Father	0	1	2	3
Mother	0	1	2	3

11、Frequently smiled at me

	very inconsistent	relatively inconsistent	relatively consistent	very consistent
Father	0	1	2	3
Mother	0	1	2	3

12、Did not seem to understand what I needed or wanted

	very inconsistent	relatively inconsistent	relatively consistent	very consistent
Father	0	1	2	3
Mother	0	1	2	3

13、Let me decide things for myself

	very inconsistent	relatively inconsistent	relatively consistent	very consistent
Father	0	1	2	3
Mother	0	1	2	3

14、Made me felt I was not wanted

	very inconsistent	relatively inconsistent	relatively consistent	very consistent
Father	0	1	2	3
Mother	0	1	2	3

15、Could make me feel better when i was upset

	very inconsistent	relatively	relatively	very consistent

		inconsistent	consistent	
Father	0	1	2	3
Mother	0	1	2	3

16、Did not talk with me very much.

	very inconsistent	relatively inconsistent	relatively consistent	very consistent
Father	0	1	2	3
Mother	0	1	2	3

17、Tried to make me feel dependent on her/him

	very inconsistent	relatively inconsistent	relatively consistent	very consistent
Father	0	1	2	3
Mother	0	1	2	3

18、Felt I could not look after myself unless she/he was around

	very inconsistent	relatively inconsistent	relatively consistent	very consistent
Father	0	1	2	3
Mother	0	1	2	3

19、Gave me as much freedom as I wanted

	very inconsistent	relatively inconsistent	relatively consistent	very consistent
Father	0	1	2	3
Mother	0	1	2	3

20、Let me go out as often as I wanted

	very inconsistent	relatively inconsistent	relatively consistent	very consistent
Father	0	1	2	3
Mother	0	1	2	3

21、Was overprotective of me

	very inconsistent	relatively inconsistent	relatively consistent	very consistent
Father	0	1	2	3
Mother	0	1	2	3

22、Did not praise me

	very inconsistent	relatively inconsistent	relatively consistent	very consistent
Father	0	1	2	3
Mother	0	1	2	3

23、Let me dress in any way I pleased

	very inconsistent	relatively inconsistent	relatively consistent	very consistent
Father	0	1	2	3
Mother	0	1	2	3

Index of well-being

Guidance: Please read each item below carefully, the numbers 1-7 represent 7 levels between the two extremes, please select a level value that suits you according to your current situation and hit " √".									
1	Tired	1	2	3	4	5	6	7	Interesting
2	Miserable	1	2	3	4	5	6	7	Happy
3	Useless	1	2	3	4	5	6	7	Valuable
4	Alone	1	2	3	4	5	6	7	Have a lot of friends
5	Empty	1	2	3	4	5	6	7	Substantial
6	Hopeless	1	2	3	4	5	6	7	Hopeful
7	Depressed	1	2	3	4	5	6	7	Have a reward
8	Life didn't give me a chance	1	2	3	4	5	6	7	Life has been so good to me
9	I am very dissatisfied with life in general	1	2	3	4	5	6	7	I am generally satisfied with my life

General Self-Efficacy Scale

Guidance: Please read each sentence below carefully. According to your current situation, choose the closest value for each question and hit "√". 1 is completely incorrect, 2 is slightly correct, 3 is mostly correct, and 4 is completely correct.					
	Items	completely incorrect	slightly correct	mostly correct	completely correct
1	I can always manage to solve difficult problems if I try hard enough.。	1	2	3	4
2	If someone opposes me, I can find the means and ways to get what I want.	1	2	3	4
3	It is easy for me to stick to my aims and accomplish my goals.	1	2	3	4
4	I am confident that I could deal efficiently with unexpected events.	1	2	3	4
5	Thanks to my resourcefulness, I know how to handle unforeseen situations.	1	2	3	4
6	I can solve most problems if I invest the necessary effort.	1	2	3	4
7	I can remain calm when facing difficulties because I can rely on my coping abilities.	1	2	3	4
8	When I am confronted with a problem, I can usually find several solutions.	1	2	3	4
9	If I am in trouble, I can usually think of a solution.	1	2	3	4
10	I can usually handle whatever comes my way.	1	2	3	4
